# Development of a character qualities test for medical students in Korea using polytomous item response theory and factor analysis: a preliminary scale development study

**DOI:** 10.3352/jeehp.2023.20.20

**Published:** 2023-06-26

**Authors:** Yera Hur, Dong Gi Seo

**Affiliations:** 1 Institute of Medical Education, Hallym University College of Medicine, Chuncheon, Korea; 2 Department of Psychology, Hallym Applied Psychology Institute, College of Social Science, Hallym University, Chuncheon, Korea; Hallym University, Korea

**Keywords:** Communication, Empathy, Leadership, Respect, Psychometrics

## Abstract

**Purpose:**

This study aimed to develop a test scale to measure the character qualities of medical students as a follow-up study on the 8 core character qualities revealed in a previous report.

**Methods:**

In total, 160 preliminary items were developed to measure 8 core character qualities. Twenty questions were assigned to each quality, and a questionnaire survey was conducted among 856 students in 5 medical schools in Korea. Using the partial credit model, polytomous item response theory analysis was carried out to analyze the goodness-of-fit, followed by exploratory factor analysis. Finally, confirmatory factor and reliability analyses were conducted with the final selected items.

**Results:**

The preliminary items for the 8 core character qualities were administered to the participants. Data from 767 students were included in the final analysis. Of the 160 preliminary items, 25 were removed by classical test theory analysis and 17 more by polytomous item response theory assessment. A total of 118 items and sub-factors were selected for exploratory factor analysis. Finally, 79 items were selected, and the validity and reliability were confirmed through confirmatory factor analysis and intra-item relevance analysis.

**Conclusion:**

The character qualities test scale developed through this study can be used to measure the character qualities corresponding to the educational goals and visions of individual medical schools in Korea. Furthermore, this measurement tool can serve as primary data for developing character qualities tools tailored to each medical school’s vision and educational goals.

## Graphical abstract

[Fig f2-jeehp-20-20]

## Introduction

### Background/rationale

The importance of character education in medical education has long been an issue. Studies on professors and students [1,2] have reported negative perceptions about whether character education in medical education is adequately implemented. Doctors in society require medical knowledge and skills and high standards of ethics, responsibility, and morality. As a result of a survey of medical education experts in the study of Hur [2], the character qualities required for medical students are defined as follows: education that fosters the basic qualities and ability to empathize with patients affected by illness based on respect for patients and others, to have basic ethical awareness and responsibility for human life, and to cooperate and communicate with colleagues.

In order to achieve practical and effective character education for medical students, rather than formal character education, educational methods and evaluation methods must be developed and applied [1,3]. To evaluate character qualities, it is necessary to develop an appropriate tool. Character qualities, which are psychological characteristics of human beings, are difficult to observe or measure directly. Self-report tests are the most frequently used method to measure various personality traits. These tests require less time, effort, and cost than other methods, and it gives respondents the advantage that they can easily express their thoughts and expressions. This method also provides an opportunity for self-evaluation and reflection in answering each question. Therefore, self-report tests can be used as helpful character evaluation tools because they allow a relatively accurate estimation of behaviors and their changes compared to face-to-face interviews [4].

In the field of medical education, item analysis research using the Rasch model is well-known [5]. In Korean medical education, there have been studies on item parameter estimation using item response theory (IRT) for medical licensing examinations [6,7]. IRT estimates the potential nature of a subject based on unique item trait curves for each item constituting the test. It is generally applied to tests that measure cognitive traits, but IRT has recently been applied to developing self-report tools to measure psychological traits [8]. In this study, we intended to develop a self-report test scale that can measure medical students’ character qualities by applying the partial credit model (PCM), which is a polytomous IRT model [9]. The PCM used in this study is suitable for self-reports, such as the Likert scale. Classical test theory and traditional statistical methods, including factor analysis and reliability analysis, were also used.

### Objectives

The purpose of this study was to develop measurement scales for a character qualities test that can be used in the field of medical education by exploring the constituent factors of 8 character qualities—namely, service and sacrifice, patience and leadership, honesty and humility, empathy and communication, responsibility and calling, care and respect, collaboration and magnanimity, and, creativity and positivity (hereinafter SPHER3C). These SPHER3C qualities were identified in our previous Delphi study [1]. To this end, first, the conceptualization and constituent factors of the SPHER3C qualities were explored; second, items that can measure the SPHER3C qualities were developed; and third, the reliability and validity of the developed items were verified.

## Methods

### Ethics statement

This study was approved by the Institutional Review Board of Hallym University (HIRB-2018-049-2-CC). Written informed consent was obtained from all participants.

### Study design

This scale development study was described according to the STROBE (Strengthening the Reporting of Observational studies in Epidemiology) statement, available from: https://www.strobe-statement.org/.

### Setting

The SPHER3C qualities required for medical students were already extracted through a Delphi survey [1]. The authors developed 20 preliminary questions for each of the SPHER3C qualities, adding up to 160 preliminary questions. During the development of the 160 preliminary questions, they were reviewed by 2 authors to confirm that they satisfactorily expressed the definition of each construct in order to ensure content validity. Five out of 40 medical schools in Korea were selected through judgmental sampling, also considering the medical school’s location and type (public or private). Students enrolled in the 5 medical schools were the study participants, who responded to the preliminary questions developed by the authors. The final items were selected by analyzing the response data through IRT and factor analysis.

### Participants

A preliminary survey was conducted targeting 856 medical students in Korea from 5 medical schools. The inclusion criteria were all target students in the 5 medical schools. There were no exclusion criteria. Data from 767 people were analyzed, excluding insincere responses. The academic level and gender distribution of the survey participants are shown in Table 1.

### Variables

The definitions and sub-qualities of the SPHER3C required for medical students are shown in Table 2. To measure these qualities, 160 preliminary questions were developed (20 questions for each SPHER3C quality).

### Data sources/measurement

To measure the SPHER3C qualities, a tool was developed as a 5-point Likert scale self-reported test with options including “strongly disagree”=1, “disagree”=2, “average”=3, “agree”=4, and “strongly agree”=5. To verify the validity and reliability of the 160 preliminary questions, an offline paper-and-pencil test was conducted from September to December 2019, targeting 856 Korean medical students from the 5 medical schools.

### Bias

Students participated in the survey voluntarily; therefore, this study did not have a randomized sample.

### Study size

For IRT, 767 examinees were enough to measure the latent traits of the examinees [9].

### Statistical methods

**Fig. 1.**
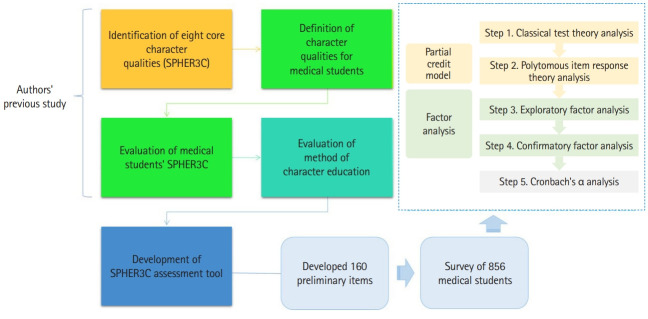
Study design and analytic process. SPHER3C, service and sacrifice, patience and leadership, honesty, and humility, empathy and communication, responsibility and calling, care and respect, collaboration and magnanimity, creativity and positivity.

As shown in Fig. 1, 5 significant data analysis steps were conducted. To develop a scale that measures the SPHER3C qualities required of medical students, preliminary questions were developed, and the final scale was constructed through the analysis of data obtained from a preliminary survey. To construct the final scale, the R program (https://www.r-project.org/) was used to select items based on classical test theory. Each of the SPHER3C qualities was first selected based on the correlation criterion between the total scores of the items, and then the response distribution of each question was checked to remove additional items that did not have responses of “strongly disagree (=1)” or “strongly agree (=5).”

Through this process, 136 out of 160 items were initially selected. For the first selected items, the DETECT index [10], a single-dimensional test based on IRT, was calculated for each character quality. Among the initially selected items, the R package ‘mirt’ (https://www.r-project.org/) was used for each character quality [11]. In addition, a multi-IRT analysis was conducted to select items secondarily based on the severity, discrimination, and agreement of each item. In the secondary selection, the infit and outfit indices were used to evaluate the agreement of the items. For the secondarily selected items, exploratory factor analysis was conducted using R (https://www.r-project.org/), and after the final item selection was completed, confirmatory factor analysis was performed using Mplus ver. 8.3 (Muthén & Muthén). Furthermore, the reliability analysis and discrimination analysis of each character quality were conducted. These 5 analytical steps are described in detail below:

Step 1. First, for the primary item selection, items were selected based on the item-total score correlation, which is used to measure the degree of discrimination in classical test theory. For the item-total score correlation, a score of 0.30 or higher was considered appropriate [12], but only items with a score of 0.2 or higher were selected in consideration of the screening procedure that would be performed later. Then, the response distribution of each item was checked, and items with very low severity due to no responses of “strongly disagree (=1)” and items with very high severity due to no responses of “strongly agree (=5)” were also removed because those items did not convey meaningful information about the participants.

Step 2. Before the secondary item selection, after confirming whether the selected items had unidimensionality, polytomous IRT analysis was conducted. The PCM used in this study is a representative polytomous IRT model. Each item’s boundary parameters and item agreement were checked, including the infit and outfit agreement [9]. Although various standards can be established according to the validation process for each item, items with a score of around 1 point are judged to be good [13]. In this analysis, items with infit and outfit indices of 0.7 or more and less than 1.2 were selected as items with good item agreement.

Step 3. Exploratory factor analysis was conducted for item selection. Kaiser-Meyer-Olkin (KMO) values and Bartlett’s sphericity test values were examined to verify the application of exploratory factor analysis. The closer the KMO value is to 1, the more appropriate the correlation of the data is for factor analysis. Usually, if it is 0.8 or higher, it is considered good, and if the Bartlett sphericity test is rejected, it means that there is a common factor in the data. The maximum likelihood method was used for exploratory factor analysis, and for the factor rotation method, Geomin rotation, which is an oblique rotation method, was mainly used. For the “honesty and humility” character quality, where each sub-factor is judged to be independent, varimax rotation, which is a direct rotation method, was applied. The final items were chosen for factor selection by checking whether there were any items with a factor loading of 0.30 or less or a variable complexity with high factor loading across several factors.

Step 4. Confirmatory factor analysis was conducted on the selected items to verify the suitability of the factor structure obtained from the results of exploratory factor analysis. As for the fitness of the model, along with verification, the comparative fit index (CFI), Tucker-Lewis index (TLI), and root mean square error of approximation (RMSEA), which are less sensitive to sample size, were confirmed. In general, a CFI and TLI of 0.90 or higher can be interpreted as indicating that a model is good, and an RMSEA of 0.08 or less can be regarded as indicating a good model [14].

Step 5. Finally, Cronbach’s α was calculated to confirm the internal consistency of the items. The correlation between the total scores and items was calculated to evaluate items’ discrimination index.

## Results

Raw response data of medical students in Korea from 5 medical schools are available from Dataset 1. Data of confirmatory factor analysis coding are available from Dataset 2.

### Classical test theory item analysis

Through classical test theory analysis, 135 items were initially selected, ranging from 12 to 19 items for each quality. When examining items based on classical test theory, the number of items selected for each character quality is shown in Table 3. The first-round selection was based on item-total score correlation and response distribution for each item (Supplement 1). Using the item-total score correlation, the items were selected based on the 0.2 criterion rather than the 0.3 criterion in consideration of the multiple-item selection process to be performed subsequently. The second round selection was based on the response distribution for each item, this involved a process of checking the percentage of all people who gave responses from “strongly disagree (=1)” to “strongly agree (=5)” for each item. The severity of the item was judged to be very low or high and was removed.

### Polytomous item response theory analysis

Based on classical test theory, the PCM was used for the initially selected items to calculate the latent score. The single-dimensional test index (DETECT) was confirmed. DETECT was computed using the sirt package [15], and the mirt package [11] was used for item analysis and latent score calculation. For the DETECT index, a score of 1 or more indicates strong multidimensionality, a score of 0.4 or more and less than 1 indicates moderate multidimensionality, and a score of less than 0.2 indicates sufficient single-dimensionality. In the case of the DETECT index, negative numbers can appear, which means that the given data has unidimensionality [10].

For the initially-selected items, all the DETECT indexes were negative, indicating unidimensionality, and IRT analysis was conducted for each character quality. The PCM model selected only items with infit and outfit of 0.7 or more and less than 1.2 and good boundary parameters with ordinality (Supplement 2). The number of items selected for each character quality is shown in Table 3.

### Exploratory factor analysis

The result of the exploratory factor analysis of the SPHER3C qualities was as follows. Tables showing the exploratory factor analysis of each character quality were added as Supplement 3, and the number of items selected for each character quality is shown in Table 3. Exploratory factor analysis was conducted within each of the 8 character qualities because each character quality is known to be independent from the other.

#### Service and sacrifice

As a result of exploratory factor analysis on 15 items for “service and sacrifice” after 2 rounds of screening, 1 factor with an eigenvalue of 1 or more was extracted from the scree plot. Four factors were extracted based on parallel analysis. However, based on the interpretability of the factors and the clarity of the factor structure, selecting 2 factors could be interpreted more clearly. The items with redundant loadings were removed, and the final 10 items were selected.

#### Patience and leadership

We conducted an exploratory factor analysis on 17 items for “patience and leadership” that went through 2 rounds of item selection, and 2 factors with an eigenvalue of 1 or more were extracted. In addition, when a parallel analysis was performed, five factors were extracted. Based on these results, the 2-factor structure was appropriate in terms of the interpretability of the factors and the clarity of the factor structure. Therefore, when the number of factors was specified and analyzed as 2 factors, and the results were confirmed, the final 10 items were selected by removing items with low factor loading and items with high variable complexity.

#### Honesty and humility

Twelve items were selected for “honesty and humility” through 2 rounds of review. As a result of exploratory factor analysis, 1 factor with an eigenvalue of 1 or more was extracted, and 4 factors were extracted through parallel analysis. However, in terms of the interpretability of the factors and the clarity of the factor structure, the 2-factor structure was appropriate. Therefore, the number of factors was designated and analyzed as 2, and the final 9 items were selected by removing 3 items with low factor loadings.

#### Empathy and communication

After 2 rounds of item selection, exploratory factor analysis was conducted on 16 items for “empathy and communication.” One factor with an eigenvalue of 1 or more was extracted, and 4 factors were extracted based on parallel analysis. However, since the interpretation of the 2-factor structure is clear, the analysis was conducted with 2 factors. Among the 16 items, cases with low factor loadings or high variable complexity were removed to select the final 10 items.

#### Responsibility and calling

“Responsibility and calling” items were selected through 2 reviews of 13 items. As a result of exploratory factor analysis, 2 factors with an eigenvalue of 1 or more were extracted, and 3 factors were extracted when parallel analysis was performed. The 2-factor structure was appropriate regarding the interpretability of the 2-factor and 3-factor structures and the clarity of the factor structure. Therefore, the number of factors was designated and analyzed as 2, and the final 10 items were selected by removing 3 items with low factor loadings or high variable complexity.

#### Care and respect

After 2 rounds of item screening, 11 items were selected for “care and respect.” Through the second item screening and as a result of exploratory factor analysis, 1 factor with an eigenvalue of 1 or more was extracted, and 4 factors were extracted as a result of the parallel analysis. However, considering the possibility of interpretability, the exploratory factor analysis was conducted based on the 2 factors because a good factor analysis was possible for the 2 factors. The final 10 items were selected after removing the items with low factor loadings.

#### Collaboration and magnanimity

For “collaboration and magnanimity,” 15 items were selected through 2 reviews, and as a result of exploratory factor analysis, 2 factors with an eigenvalue of 1 or more were extracted. Four factors were extracted as a result of the parallel analysis. Considering these results, the number of factors was selected as 2 based on the interpretability of the factors and the clarity of the factor structure. The final 10 items were selected after removing items with low factor loading and high variable complexity.

#### Creativity and positivity

For “creativity and positivity,” 17 items were selected through 2 rounds of item review, and as a result of exploratory factor analysis, 2 factors with an eigenvalue of 1 or more were extracted. Four factors were extracted as a result of the parallel analysis. Here, the number of factors was selected as 2, based on the interpretability of the factors and the clarity of the factor structure. Among the 17 items, no items with factor loadings of 0.30 or less were found, but items with factor loadings of 0.40 or less were removed to compose items with a structure similar to other factors. Furthermore, items with variable complexity or low factor loadings were removed, resulting in 10 final items.

### Confirmatory factor analysis

Confirmatory factor analysis was conducted to determine whether it was appropriate to construct a tool to measure the 8 SPHER3C qualities with a factor structure obtained through exploratory factor analysis. As shown in Table 4 and Supplement 4, the model’s goodness of fit was found to be appropriate. Only the “honesty and humility” quality had a CFI and TLI that were less than 0.90, and RMSEA was above 0.80, indicating the poor fit.

### Reliability analysis

Cronbach’s α values for the SPHER3C factors ranged between 0.637 and 0.784 for each sub-factor (Table 5). Sub-factors with final selected items showed good internal consistency. In addition, to collect basic information for evaluating the quality of each item, the item-total correlation (item discrimination index) was calculated. As a result, the total score-item correlations for all sub-factors were higher than 0.30.

### Final items selected for the SPHER3C test

Supplement 5 shows the 79 final items of the scale in Korean SPHER3C qualities of the medical students. The English version of the final items can be found in Supplement 3.

## Discussion

### Key results

In order to develop a character quality test for medical students, 160 preliminary questions were developed according to the sub-qualities and definitions of the SPHER3C qualities. We analyzed the data obtained from the primary test tool for Korean medical students. To develop the final tool, 81 items were removed by applying classical test theory, PCM in polytomous IRT, and exploratory factor analysis to select the final items and sub-factors. A total of 79 final items were selected, and the validity and reliability of the items were confirmed through confirmatory factor analysis for each of the SPHER3C factors and intra-item relevance analysis.

### Interpretation

In the past, there have been studies on character qualities in medical students or the development of tools to measure medical professionalism. However, there has been no study measuring the character qualities of Korean medical students. The strength of this study lies here; consequently, it is difficult to compare this study with the results of other studies as there are no previous studies for comparison.

The final test to measure the character qualities of medical students consisted of 8 character qualities (SPHER3C), 16 sub-factors, and 79 items. The final test was constructed to measure 10 items for each quality, except for “honesty and humility” quality, for which we could only extract 9 items.

The validity of the final test was confirmed through confirmatory factor analysis of the items and factor structure selected through PCM and exploratory factor analysis. All showed a good fit, meeting the corresponding criteria. The Cronbach’s α coefficient of the 79 finally selected questions was 0.929, indicating high reliability.

### Limitations and suggestions

The limitations of this study and suggestions for follow-up studies are as follows.

First, this study’s character qualities test was written in Korean. When using translated items in another language, the items must reflect the social and cultural differences of the region where the test will be conducted. It also must be determined that the translation is similar to what the original test intends to measure by conducting measurement equivalence verification. For “collaboration and magnanimity,” only the reverse-scored items were selected as the items for the inclusion factor. However, since the item-total correlation was positive, this did not appear to be a reverse scoring problem. This may have been because there were too many grading questions that students did not mark carefully. Alternatively, unlike English, Korean-language responses to negative sentences may not be clear.

Second, this study analyzed data obtained through 160 preliminary items, extracting 79 items. A follow-up study for data collection and verification of the finally constructed test with 79 items would be needed. In particular, it is necessary to verify test-retest reliability and accreditation validity.

Third, the character qualities test questions developed in this study were not designed as questions in a medical situation. This was to allow first-year students with no medical education background to take the test, since we wanted a tool that could be taken for all medical students regardless of their academic level. However, to evaluate character qualities in a specific situation, it is necessary to develop a situational judgment test in addition to a self-reported measure or a test that applies behavioral anchored rating scales (BARS) instead of a Likert scale. Although self-reported tests are valuable tools for character measurement, they also have limitations. A situational judgment test or BARS scale can supplement the limitations of self-report tests.

### Conclusion

To develop a test to measure the SPHER3C factors in medical students, the PCM can be applied through IRT. The quality of the character qualities evaluation tool could be improved by applying goodness-of-fit tests for item selection. In addition, the tool’s validity was ensured by using factor analysis, a traditional statistical method, during test development. The SPHER3C test can be used to measure the character quality factors corresponding to the educational goals and talents of each university in Korea and utilized as primary data for developing a character qualities measurement tool tailored to each university’s vision and educational goals.

## Figures and Tables

**Figure f2-jeehp-20-20:**
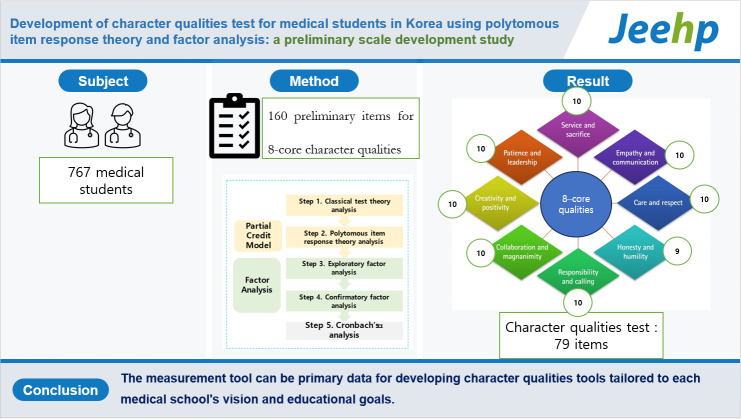


**Table 1.** Academic-year and gender-based distribution of the participants

Classification	Frequency (%)
Gender	
Male	481 (62.7)
Female	279 (36.4)
No indication	7 (0.9)
Total	767 (100.0)
Academic level	
Year 1	130 (16.9)
Year 2	878 (11.5)
Year 3	315 (41.1)
Year 4	119 (15.5)
Year 5	81 (10.6)
Year 6	34 (4.4)
Total	767 (100.0)

**Table 2.** Definition and sub-elements of 8 core character qualities

Core character qualities	Definition	Sub-qualities
Service and sacrifice	The attitude of thinking of others (patients) before one’s own personal interests, sacrificing oneself for others, devoting oneself to society, and practicing volunteer work through medical practice	Service, sacrifice, dedication, devotion, altruistic attitude, warmth, concession, fraternity, appreciation
Patience and leadership	Attitudes and ability to reflect on, examine, and endure difficult situations, to view health care in its social context, and to reach an agreement with other members of an organization	Patience, leadership, self-reflection, self-identity, social cognitive ability
Honesty and humility	Being faithful or honest to yourself or others in a straightforward way, without lies or deception, without being arrogant or ignorant of others, and knowing how to act in a humble way	Honesty, diligence, humility, ethical judgment, morality, conscience, moral judgment, authenticity
Empathy and communication	Attitude and ability to interact and communicate well while accurately communicating thoughts and emotions, knowing how to understand and sympathize with others’ thoughts, feelings, and perspectives	Communication skills, empathy, expressive power, conflict management, listening, sincerity
Responsibility and calling	The intention of fulfilling one’s tasks faithfully and responsibly, protecting the fundamental rights and human rights of patients, appreciating the doctor’s profession, and contributing to society through the profession	Responsibility, medical ethics, accountability, calling, sense of duty
Care and respect	Acting in consideration of the position of others, understanding and respecting other positions, respecting the noble nature of life, being attentive to care for others, and caring for others	Consideration, respect for people, respect for life, kindness, tolerance
Collaboration and magnanimity	Attitude and ability to be interested in group and community issues, interacting with members, and working together to achieve common goals	Cooperation, an embracing spirit, community, mutual exchange, interdependence
Creativity and positivity	The attitude of not being confined to existing frameworks but being able to look at things and situations with new and open eyes, and seeking various ways to solve problems with good results even in difficult situations	Creativity, positivity, open-mindedness, mindfulness of looking at problems from multiple angles, courage

Modified from Tables 2 and 3 from Hur & Lee. J Educ Eval Health Prof 2019;16:21 [1].

**Table 3.** Number of selected items from the first and second rounds of selection and exploratory factor analysis

Core character qualities	Preliminary items	Primary selection of items	Secondary selection of items	Selection from factor analysis
Service and sacrifice	20	19	15	10
Patience and leadership	20	17	17	10
Honesty and humility	20	12	12	9
Empathy and communication	20	18	16	10
Responsibility and calling	20	16	13	10
Care and respect	20	17	11	10
Collaboration and magnanimity	20	19	15	10
Creativity and positivity	20	17	17	10
Total	160	135	118	79

First-round selection: item-total correlation, second-round selection: item response distribution.

**Table 4.** Results of confirmatory factor analysis

Core character qualities	χ^2^ (df)	CFL	TLI	RMSEA
Service and sacrifice	103.152 (34)^*^	0.957	0.943	0.0051 (0.04–0.0063)
Patience and leadership	112.194 (34)^*^	0.930	0.907	0.055 (0.044–0.0066)
Honesty and humility	193.134 (26)^*^	0.818	0.749	0.092 (0.08–0.104)
Empathy and communication	168.432 (34)^*^	0.922	0.897	0.072 (0.061–0.083)
Responsibility and calling	98.003 (34)^*^	0.940	0.920	0.05 (0.038–0.061)
Care and respect	9.272 (34)^*^	0.962	0.949	0.046 (0.035–0.058)
Collaboration and magnanimity	112.327 (34)^*^	0.957	0.943	0.055 (0.044–0.066)
Creativity and positivity	181.509 (34)^*^	0.916	0.889	0.075 (0.065–0.086)

df, degree of freedom; CFL, comparative fit index; TLI, Tucker-Lewis index; RMSEA, root mean square error of approximation.

* P<0.05.

**Table 5.** Reliability scores of SPHER3C and sub-factors

Core character qualities	Mean±SD	ITC	Cronbach’s α
Service and sacrifice			
Service			0.720
I1	3.294±1.053	0.426	0.716
I17	3.873±0.804	0.495	0.682
I33	3.855±0.781	0.648	0.632
I41	3.619±0.877	0.506	0.675
I129	3.661±0.780	0.561	0.660
Sacrifice			0.726
I49	3.520±0.882	0.472	0.697
I73	3.455±0.839	0.528	0.679
I81	3.154±0.969	0.534	0.671
I89	3.020±0.948	0.49	0.689
I97	3.267±0.977	0.559	0.661
Total	3.472		0.801
Patience and leadership			
Patience			0.723
I24	3.402±0.967	0.628	0.634
I32	3.142±1.102	0.523	0.669
I40	2.807±1.039	0.464	0.691
I128	2.737±1.020	0.402	0.714
I160	3.253±0.984	0.533	0.667
Leadership			0.61
I64	3.675±0.841	0.284	0.604
I72	3.617±0.885	0.436	0.534
I112	3.706±0.932	0.407	0.547
I144	3.736±0.804	0.470	0.525
I152	3.390±0.975	0.369	0.567
Total	3.350		6.570
Honesty and humility			
Humility			0.560
I108	4.150±0.732	0.287	0.605
I132	3.190±1.061	0.447	0.363
I156	3.570±1.017	0.478	0.321
Honesty			0.655
I12	3.955±0.778	0.52	0.584
I84	3.853±0.775	0.469	0.600
I100	3.628±0.838	0.403	0.617
I116	3.503±0.812	0.359	0.630
I124	3.009±1.071	0.366	0.635
I140	3.560±0.854	0.411	0.613
Total	3.602		0.622
Empathy and communication			
Communication			0.800
I34	3.513±0.924	0.637	0.757
I50	3.430±0.997	0.660	0.750
I58	3.838±0.763	0.580	0.778
I138	3.783±0.768	0.614	0.769
I154	3.685±0.822	0.655	0.754
Empathy			0.640
I2	3.898±0.774	0.368	0.613
I66	3.728±0.758	0.525	0.543
I106	3.831±0.731	0.430	0.586
I114	3.686±0.826	0.366	0.613
I122	3.692±0.813	0.457	0.570
Total	3.708		0.790
Responsibility and calling			
Calling			0.711
I13	2.650±0.971	0.443	0.683
I21	2.761±0.874	0.526	0.652
I69	2.971±0.916	0.559	0.637
I85	2.896±0.989	0.48	0.668
I133	2.847±0.918	0.468	0.672
Responsibility			0.632
I5	3.827±0.937	0.390	0.592
I29	3.910±0.863	0.431	0.573
I37	3.556±0.891	0.494	0.538
I53	4.144±0.734	0.404	0.592
I77	3.294±1.009	0.375	0.598
Total	3.286		0.654
Care and respect			
Care			0.700
I67	3.827±0.770	0.527	0.647
I75	3.959±0.681	0.517	0.656
I123	3.915±0.706	0.525	0.651
I131	3.788±0.755	0.461	0.672
I155	4.149±0.714	0.527	0.650
Respect			0.700
I3	4.294±0.725	0.540	0.647
I19	3.938±0.689	0.457	0.673
I27	4.222±0.729	0.582	0.627
I35	4.021±0.721	0.457	0.672
I51	4.073±0.730	0.530	0.646
Total	4.019		0.793
Collaboration and magnanimity			
Magnanimity			0.815
I14	3.441±1.041	0.467	0.823
I54	3.19±1.138	0.682	0.764
I62	3.031±1.124	0.64	0.776
I70	3.237±1.076	0.693	0.762
I78	3.366±1.132	0.698	0.761
Collaboration			0.674
I86	3.912±0.76	0.425	0.643
I102	3.959±0.765	0.497	0.616
I110	3.777±0.755	0.444	0.634
I118	3.925±0.706	0.541	0.599
I158	3.957±0.687	0.472	0.627
Total	3.580		0.768
Creativity and positivity			
Creativity			0.779
I55	3.194±0.973	0.655	0.714
I63	3.469±0.992	0.534	0.753
I71	3.315±0.959	0.449	0.778
I111	3.140±1.000	0.659	0.711
I127	3.380±0.996	0.618	0.726
Positivity			0.709
I15	3.749±0.855	0.539	0.645
I23	3.808±0.803	0.452	0.679
I31	3.905±0.770	0.534	0.652
I39	3.889±0.910	0.487	0.670
I47	3.86±0.791	0.52	0.656
Total	3.571		0.752

SPHER3C, service and sacrifice, patience and leadership, honesty and humility, empathy and communication, responsibility and calling, care and respect, collaboration and magnanimity, creativity and positivity; SD, standard deviation; ITC, item-total correlation.

## Footnotes

**Data availability**
Data files are available from Harvard Dataverse: https://doi.org/10.7910/DVN/UE55JT Dataset 1. Raw response data of medical students in Korea from 5 medical schools.

Dataset 2. Data of confirmatory factor analysis coding.

## Supplementary materials

Supplementary files are available from Harvard Dataverse: https://doi.org/10.7910/DVN/UE55JT

Supplement 1. Response distribution for each item on the SPHER3C factors.

Supplement 2. Item parameters (partial credit model) for each item on the SPHER3C factors.

Supplement 3. The exploratory factor analysis verification results of the SPHER3C qualities, including English translation of the final items for core character qualities test for medical students.

Supplement 4. The confirmatory factor analysis verification results of the SPHER3C qualities.

Supplement 5. Final items selected for core character qualities test for medical students in Korean.

Supplement 6. Audio recording of the abstract.
