# Supplementary material for: Development of a character qualities test for medical students in Korea using polytomous item response theory and factor analysis: a preliminary scale development study
Source: J Educ Eval Health Prof. 2023 Jun 26;20:20. doi: 10.3352/jeehp.2023.20.20 (PMC10356546; doi:10.3352/jeehp.2023.20.20)
Supplement: Supplementary file 6 — Supplement 4. The confirmatory factor analysis verification results of the SPHER3C qualities. [file jeehp-20-20-suppl4.docx]

Supplememt 4. The confirmatory factor analysis verification results of SPHER3C qualities


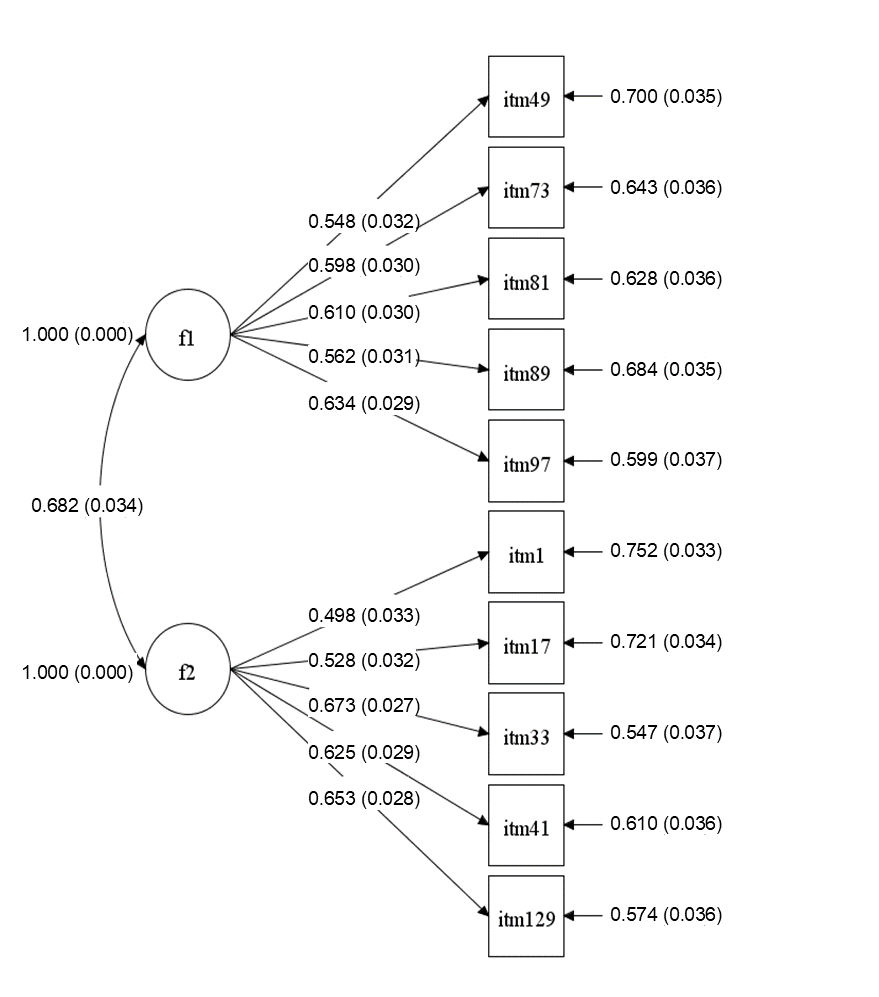


Fig 1. Confirmatory factor analysis verification result of “Service and sacrifice”


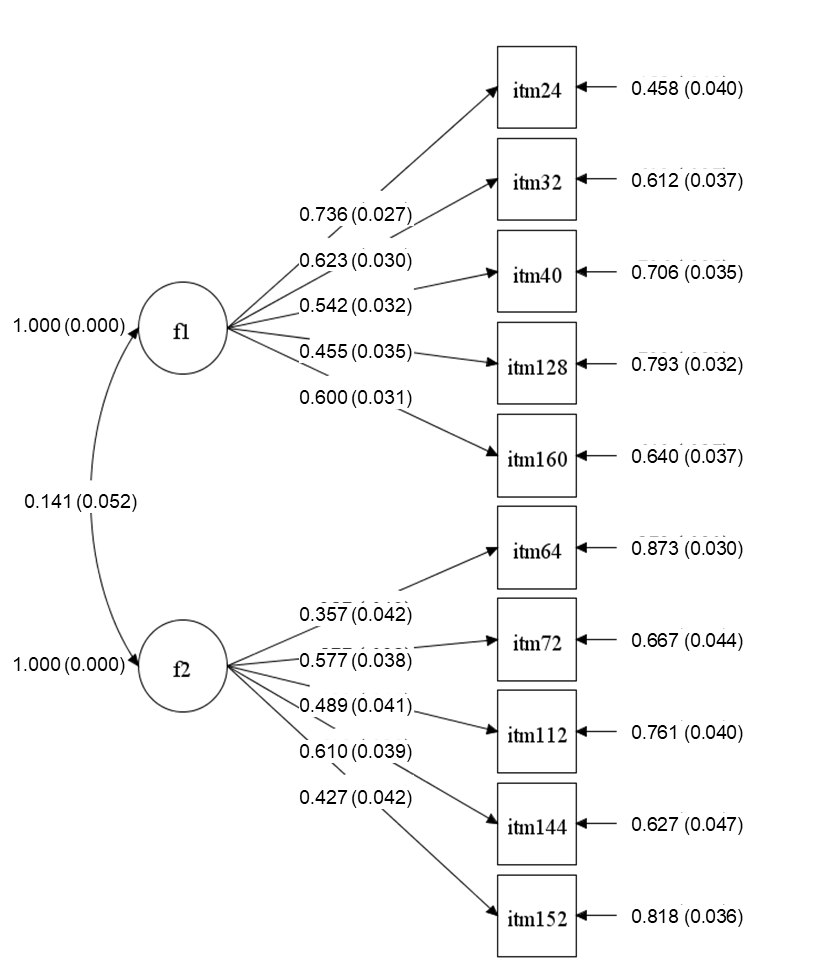


Fig 2. Confirmatory factor analysis verification result of “Patience and leadership”


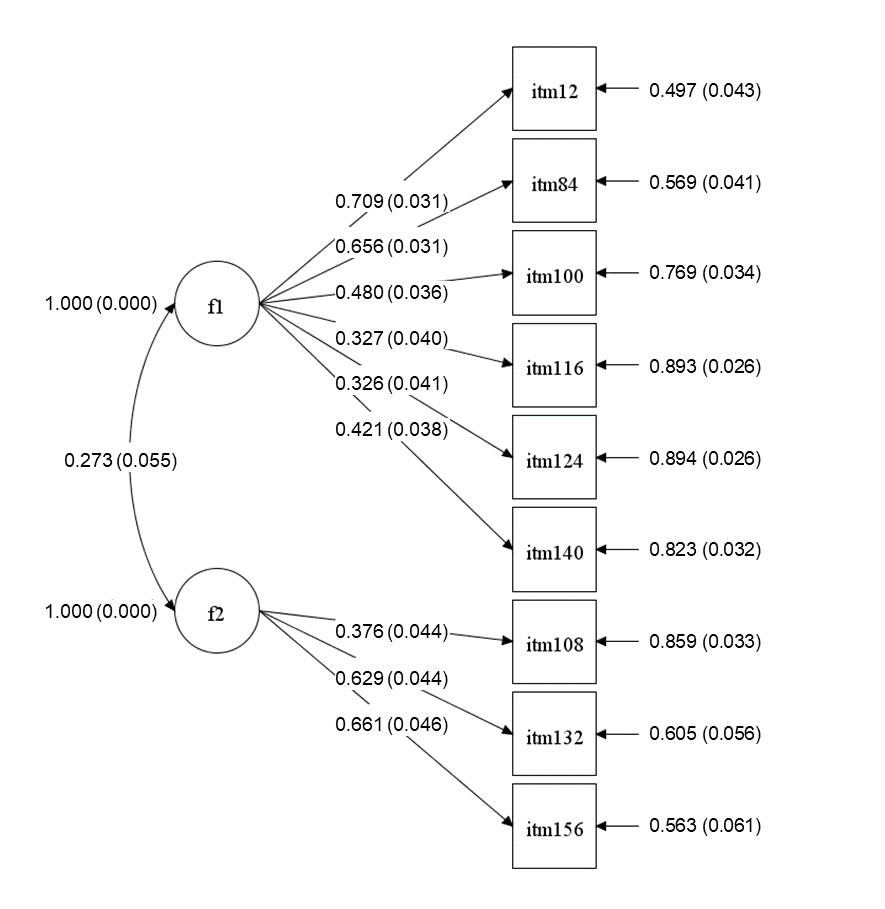


Fig 3. Confirmatory factor analysis verification result of “Honesty and humility”


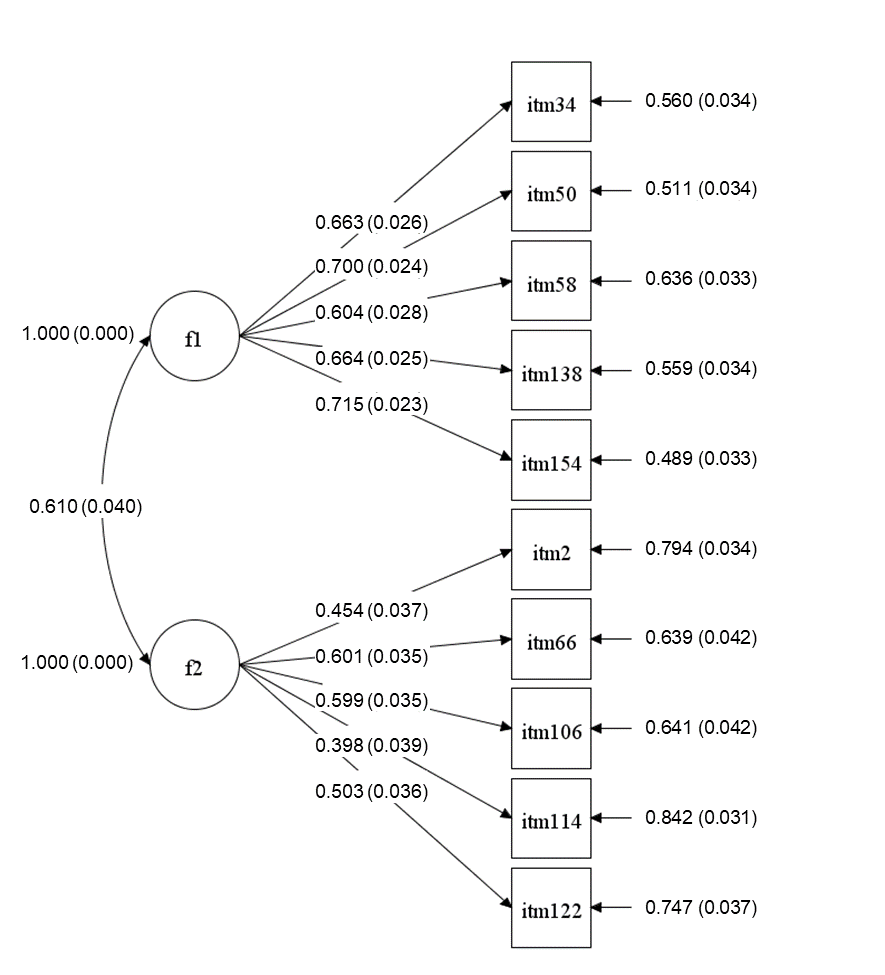


Fig 4. Confirmatory factor analysis verification result of “Empathy and communication”


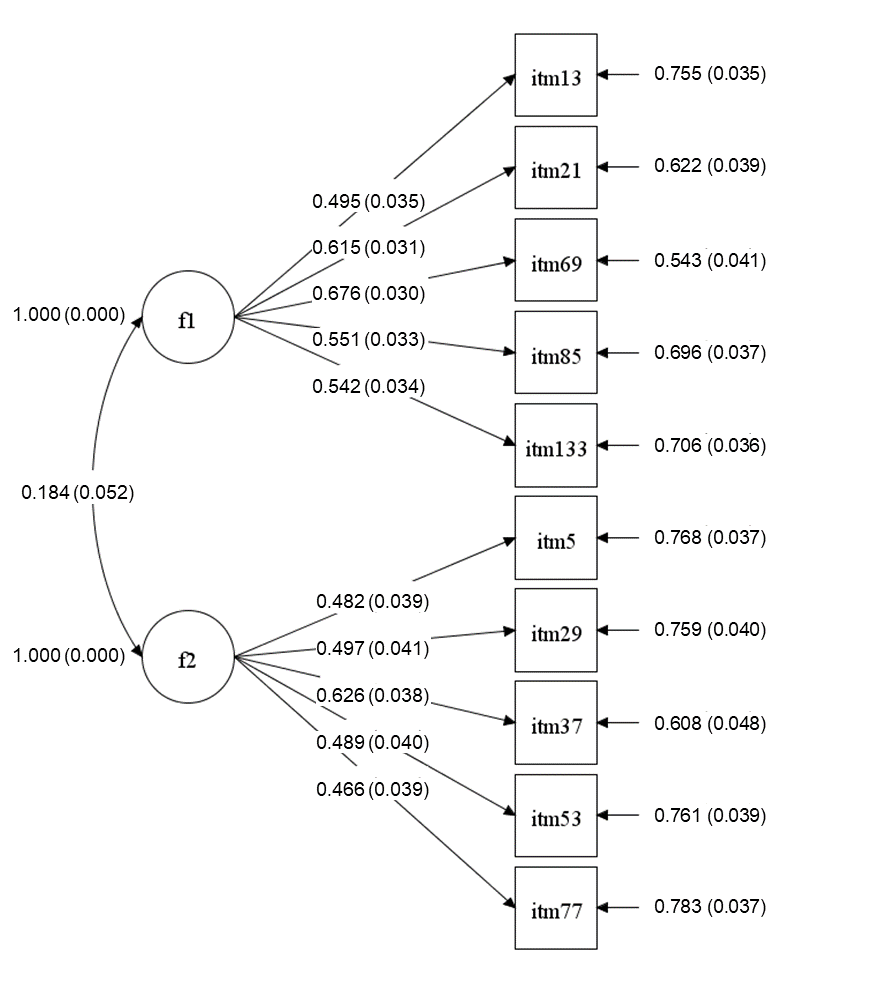


Fig 5. Confirmatory factor analysis verification result of “Responsibility and calling”


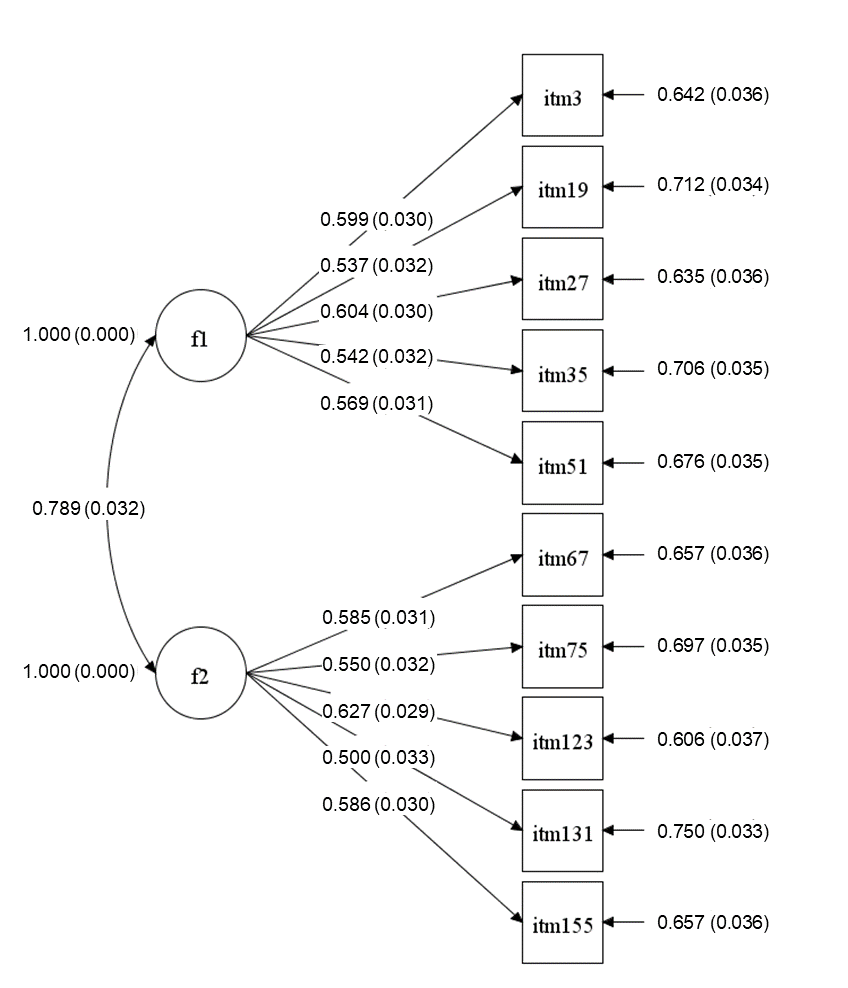


Fig 6. Confirmatory factor analysis verification result of “Care and respect”


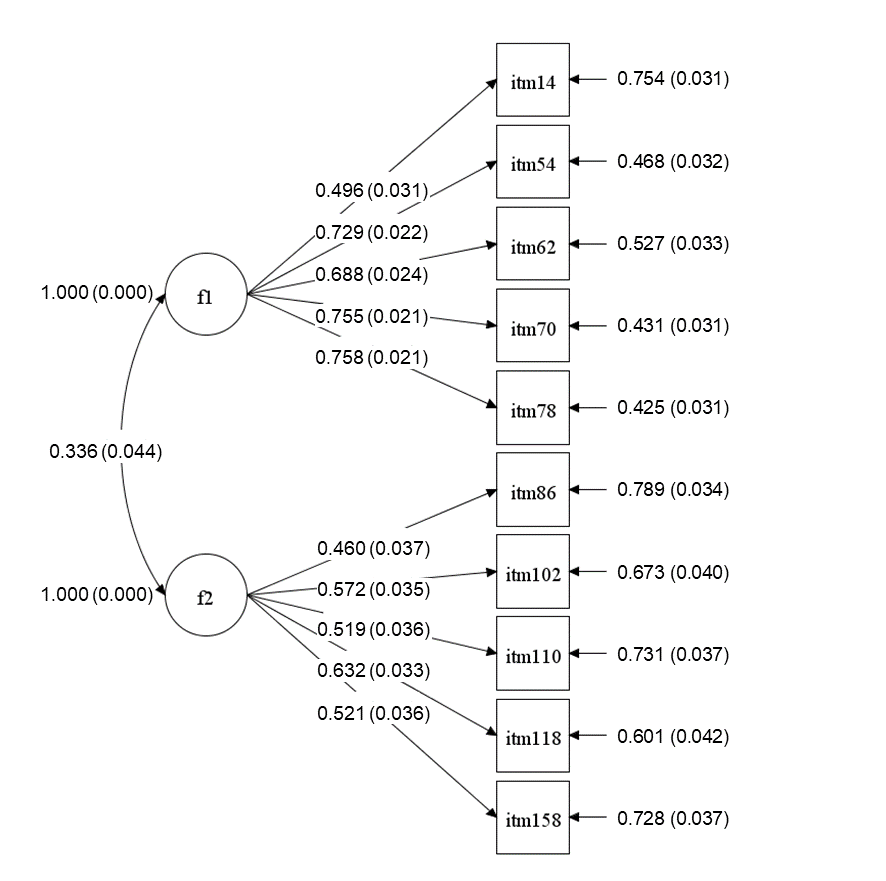


Fig 7. Confirmatory factor analysis verification result of “Collaboration and magnanimity”


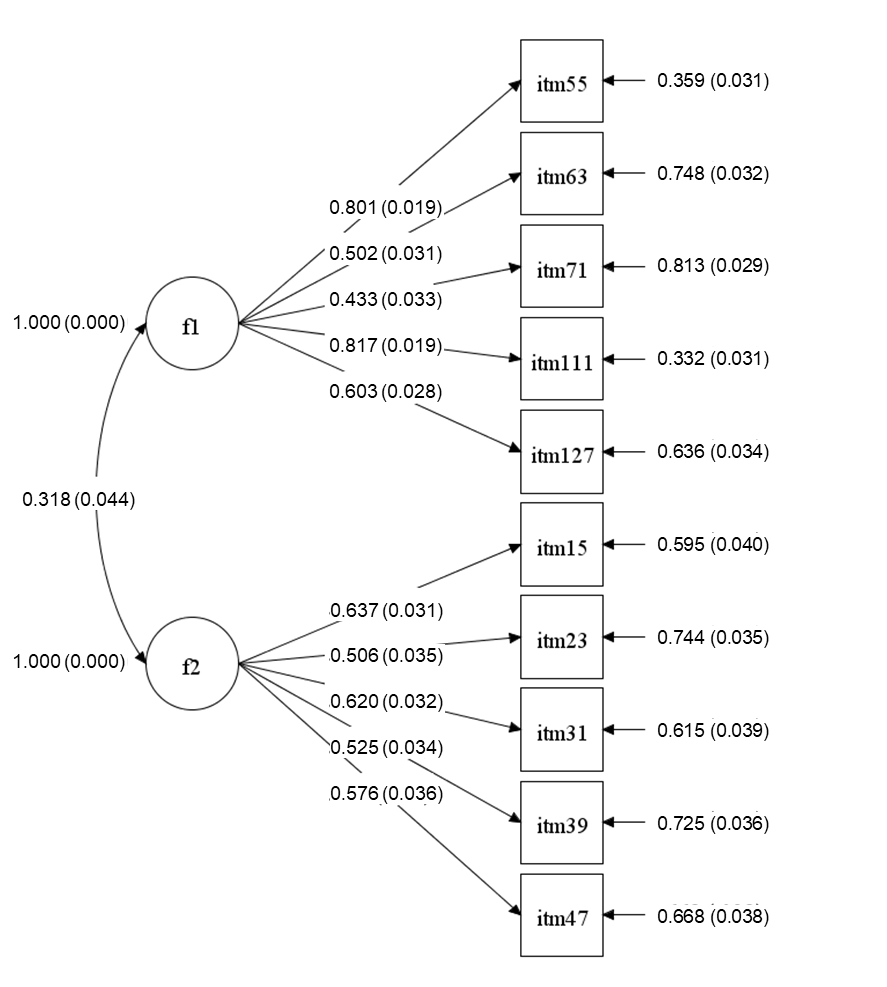


Fig 8. Confirmatory factor analysis verification result of “Creativity and positivity”
