# Supplementary material for: Development of a character qualities test for medical students in Korea using polytomous item response theory and factor analysis: a preliminary scale development study
Source: J Educ Eval Health Prof. 2023 Jun 26;20:20. doi: 10.3352/jeehp.2023.20.20 (PMC10356546; doi:10.3352/jeehp.2023.20.20)
Supplement: Supplementary file 7 — Supplement 5. Final items selected for core character qualities test for medical students in Korean. [file jeehp-20-20-suppl5.docx]

Supplement 5. Final items selected for core character qualities test for medical students

| **핵심요소** | **하위요소** | **문항** |
| --- | --- | --- |
| 봉사와 희생 | 봉사 | 봉사활동은 시민으로서의 의무라고 생각한다. |
|  |  | 사회적 약자를 도와줄 수 있는 능력을 가지고 있다. |
|  |  | 불행한 사람들을 보면 그들에게 도움이 되는 일을 하고 싶다. |
|  |  | 나는 남들보다 이타적인 마음을 가지고 있는 사람이다. |
|  |  | 다른 사람을 도와주는 일을 좋아한다. |
|  | 희생 | 내가 싫어하는 일이라도 다른 사람이 도움을 요청하면 들어 준다. |
|  |  | 나에게 확실히 이득이 될지라도 필요하다면 다른 사람을 위해 양보한다. |
|  |  | 내 일이 밀려있는 상황이라도 다른 사람이 부탁하면 도와준다. |
|  |  | 다른 사람들이 하기 싫어하는 일이 있으면 먼저 맡아서 한다. |
|  |  | 더 많은 사람들이 이익을 볼 수 있다면 내가 손해 봐도 괜찮다. |
| 인내심과 리더십 | 인내심 | 내가 생각한대로 일이 풀리지 않으면 쉽게 포기하는 편이다. |
|  |  | 다른 사람들에 비해 쉽게 지친다. |
|  |  | 다른 재미에 빠져서 할 일을 마무리하지 못하는 경우가 많다. |
|  |  | 남들 앞에 내가 먼저 나서는 경우는 거의 없다. |
|  |  | 문제가 생겼을 때 적절한 해결책을 제시하는 것이 어렵다. |
|  | 리더십 | 하고 싶은 일은 시간이 많이 걸리더라도 해내고야 만다. |
|  |  | 나는 나의 장단점을 모두 잘 알고 있다. |
|  |  | 단점을 포함하여 내 모습 그대로가 싫지 않다. |
|  |  | 친한 친구들의 장단점을 명확히 파악하고 있다. |
|  |  | 상대방에게 필요한 일을 부탁하는 것이 불편하지 않다. |
| 정직과 겸손 | 정직 | 나는 내가 잘못한 것에 대해 정직하게 시인하는 편이다. |
|  |  | 나의 실수나 잘못을 순순히 인정한다. |
|  |  | 나의 부족한 점을 남들에게 숨기지 않는다. |
|  |  | 나는 타인의 바람직하지 않는 압력에 굴복하지 않는다. |
|  |  | 나는 어떤 사람에게든 똑같이 대한다. |
|  |  | 손해를 본다고 하더라도 나에게 유리한 방향으로 상황을 꾸며내지 않는다. |
|  | 겸손 | 나는 스스로 부끄럽지 않은 사람이 되고자 노력한다. |
|  |  | 내 이익을 위해 편법을 사용할 수 있다면 그렇게 하겠다. |
|  |  | 나는 일이 잘 되지 않을 때 다른 사람 탓으로 돌린다. |
| 공감과 소통 | 공감 | 누군가의 실수를 비난하기 전에 상대의 마음을 먼저 헤아려 본다. |
|  |  | 나는 선입견을 버리고 상대방의 의견을 경청할 수 있다. |
|  |  | 나는 상대방이 이해하기 쉬운 언어로 대화할 수 있다. |
|  |  | 나는 대화할 때 상대방의 말을 끊지 않는다. |
|  |  | 나는 자신의 감정을 조절하면서 대화할 수 있다. |
|  | 소통 | 마땅히 할 이야기가 없어도 상대방과 즐겁게 대화할 수 있다. |
|  |  | 처음 보는 사람과도 쉽게 대화할 수 있다. |
|  |  | 나는 대화할 때 한 주제에서 다른 주제로 자연스럽게 넘어갈 수 있다. |
|  |  | 나는 다른 사람들과 공감대를 잘 형성하는 편이다. |
|  |  | 나는 처음 만나는 사람이라도 그 사람에 맞춰서 대화할 수 있다. |
| 책임과 소명 | 책임 | 계획한 일을 끝마치기 전에는 마음이 편하지가 않다. |
|  |  | 나 때문에 일이 그르치는 것을 보면 참을 수 없다. |
|  |  | 나는 맡은 일을 절대 대충 하는 법이 없다. |
|  |  | 내가 맡은 일을 스스로에게 부끄럽지 않게 잘 해내고 싶다. |
|  |  | 내가 하겠다고 한 일을 다른 사람에게 넘기는 것은 내 자신이 용납이 안 된다. |
|  | 소명 | 굳이 내게 주어진 일 이상을 해야 할 의무는 없다. |
|  |  | 꼭 해야 할 일이라면, 굳이 내가 아니어도 누군가가 나설 것이다. |
|  |  | 내가 안 해도 어떻게 되겠지 하는 생각이 자주 든다. |
|  |  | 단체 작업이라면 일이 잘못되어도 반드시 내 책임은 아니다. |
|  |  | 내가 잘못한 것이 있더라도 상대방이 모르면 굳이 밝힐 필요는 없다. |
| 배려와 존중 | 배려 | 나는 타인에 대한 관심과 존경을 잘 표현한다. |
|  |  | 나는 다른 사람의 부탁을 받으면 도와주는 편이다. |
|  |  | 나는 타인과 이야기할 때 상대의 마음을 생각해본다. |
|  |  | 나는 상대방이 나보다 먼저 하고 싶어 하는 말이 있는지 살핀다. |
|  |  | 나는 다른 사람에게 도움을 받으면 항상 감사를 표시한다. |
|  | 존중 | 남들은 나와 다르게 생각할 수도 있다는 것을 항상 염두에 둔다. |
|  |  | 내 생각과 반대되는 의견이 생기면 다시 한번 고민해본다. |
|  |  | 다른 사람의 생각이 내 생각과 반드시 일치할 필요는 없다 |
|  |  | 다양한 의견을 많이 접하면 더 좋은 의견이 나오게 된다 |
|  |  | 직접 경험하지 못한 일은 섣불리 판단해서는 안 된다. |
| 협력과 포용 | 협력 | 나는 타인과 대화할 때 상대방의 말을 끝까지 들어준다. |
|  |  | 나는 모르는 것이 있을 때 주변 사람들에게 도움을 요청한다. |
|  |  | 나는 내가 가진 새로운 정보나 노하우를 다른 사람과 공유한다. |
|  |  | 나는 상대방과 문제가 생겼을 때 그 원인을 찾으려고 노력한다. |
|  |  | 나는 상대방의 의견이 타당하다면 내 의견과 다르더라도 받아들인다. |
|  | 포용 | 내가 알게된 새로운 정보나 노하우를 남에게 공유하고 싶지 않다. |
|  |  | 다른 사람들과 많은 시간을 보내는 것은 내게 중요한 일이 아니다. |
|  |  | 나는 다른 사람에게 별 관심이 없다. |
|  |  | 여러 사람들과 가깝게 지내는 것은 불편하다. |
|  |  | 여러 사람들이 모이는 자리는 재미가 없다. |
| 창의성과  긍정성 | 창의성 | 남들보다 새로운 아이디어를 더 잘 생각해내는 것 같다. |
|  |  | 남들이 어려워하는 문제들을 풀어보기 좋아한다. |
|  |  | 남들이 좀처럼 관심을 두지 않는 것들에도 관심이 많다. |
|  |  | 독창적인 아이디어가 많이 떠오르는 편이다. |
|  |  | 추상적이고 복잡한 문제를 해결하는 것이 흥미롭다. |
|  | 긍정성 | 내일은 지금보다 더 나은 내가 되어있을 것이라고 생각한다. |
|  |  | 어떤 사람이나 문제에 대해서 긍정적인 면을 먼저 보려고 한다. |
|  |  | 어떤 어려운 문제라도 해결할 수 있는 실마리가 있다고 생각한다. |
|  |  | 무슨 일이든 열심히 하면 언젠가는 보상이 따라온다고 생각한다. |
|  |  | 지금 당장은 어려워도 미래를 생각하면서 열심히 살고자 노력한다. |
